# Prediction of 30-day risk of acute exacerbation of readmission in elderly patients with COPD based on support vector machine model

**DOI:** 10.1186/s12890-022-02085-w

**Published:** 2022-07-30

**Authors:** Rui Zhang, Hongyan Lu, Yan Chang, Xiaona Zhang, Jie Zhao, Xindan Li

**Affiliations:** Department of Nursing, The General Hospital of Ningxia Medical University, Yinchuan, 750004 People’s Republic of China

**Keywords:** Old age, COPD, SVM

## Abstract

**Background:**

Acute exacerbation of chronic obstructive pulmonary disease (COPD) is an important event in the process of disease management. Early identification of high-risk groups for readmission and appropriate measures can avoid readmission in some groups, but there is still a lack of specific prediction tools. The predictive performance of the model built by support vector machine (SVM) has been gradually recognized by the medical field. This study intends to predict the risk of acute exacerbation of readmission in elderly COPD patients within 30 days by SVM, in order to provide scientific basis for screening and prevention of high-risk patients with readmission.

**Methods:**

A total of 1058 elderly COPD patients from the respiratory department of 13 general hospitals in Ningxia region of China from April 2019 to August 2020 were selected as the study subjects by convenience sampling method, and were followed up to 30 days after discharge. Discuss the influencing factors of patient readmission, and built four kernel function models of Linear-SVM, Polynomial-SVM, Sigmoid-SVM and RBF-SVM based on the influencing factors. According to the ratio of training set and test set 7:3, they are divided into training set samples and test set samples, Analyze and Compare the prediction efficiency of the four kernel functions by the precision, recall, accuracy, F1 index and area under the ROC curve (AUC).

**Results:**

Education level, smoking status, coronary heart disease, hospitalization times of acute exacerbation of COPD in the past 1 year, whether long-term home oxygen therapy, whether regular medication, nutritional status and seasonal factors were the influencing factors for readmission. The training set shows that Linear-SVM, Polynomial-SVM, Sigmoid-SVM and RBF-SVM precision respectively were 69.89, 78.07, 79.37 and 84.21; Recall respectively were 50.78, 69.53, 78.74 and 88.19; Accuracy respectively were 83.92, 88.69, 90.81 and 93.82; F1 index respectively were 0.59, 0.74, 0.79 and 0.86; AUC were 0.722, 0.819, 0.866 and 0.918. Test set precision respectively were86.36, 87.50, 80.77 and 88.24; Recall respectively were51.35, 75.68, 56.76 and 81.08; Accuracy respectively were 85.11, 90.78, 85.11 and 92.20; F1 index respectively were 0.64, 0.81, 0.67 and 0.85; AUC respectively were 0.742, 0.858, 0.759 and 0.885.

**Conclusions:**

This study found the factors that may affect readmission, and the SVM model constructed based on the above factors achieved a certain predictive effect on the risk of readmission, which has certain reference value.

## Background

Chronic obstructive pulmonary disease (COPD) is a disease that seriously endangers human health, causing huge socioeconomic and health burdens worldwide, and has become a major challenge to public health [1, 2]. According to the Global Initiative on Chronic Obstructive Pulmonary Disease (GOLD)(2021): acute exacerbation of chronic obstructive pulmonary disease is an important event in the process of disease management [3]. Due to the enhanced chronic inflammatory response of the airway and lungs to toxic particles or gases, airflow restriction is often progressive in COPD patients, and acute exacerbations occur repeatedly in the course of disease development [1]. Studies have shown that the readmission rate of COPD patients with acute exacerbation within 30 days was 6.70–22.60%, and acute exacerbation readmission within 30 days due to its short acute exacerbation cycle not only seriously damages lung function and increases the risk of death, but also occupies a large number of medical resources [4].

Research shows that early identification of high-risk populations for readmission and appropriate measures can prevent readmission in some populations [5]. Lau et al. [6] used the RACE scale and LACE to predict the readmission of COPD patients in the United States, Australia and other countries, and tested their effectiveness. Donze et al. [7] used the HOSPITAL scale to predict readmission in the US and even international multicenter populations. In addition, some scholars have predicted and compared the risk of acute exacerbation readmission within 30 days of COPD patients based on accelerometer-based activity monitoring, predictive scales and related models [8–11]. The results of the studies all show that the risk prediction results based on the model are more reliable, but it is still inconclusive which way the model constructed is more suitable for the risk screening of readmission. Although Goto et al. [5] compared logistic regression model with decision tree algorithm model and deep neural network model, they still lacked specific prediction tools. As an emerging model building method, support vector machine (SVM) is an important technology in data mining classification, and its predictive performance has been gradually recognized by the medical field, but there is still a lack of relevant reports in the risk prediction of readmission in COPD patients [12].

Our study assumes that the SVM model can achieve a certain prediction effect in predicting the risk of readmission in COPD patients, and the results have certain reference value. Therefore, it is proposed to use SVM to build a 30-day acute exacerbation readmission risk prediction model for elderly COPD patients, and evaluate its prediction effect, so as to provide a basis for early identification of patients with high risk of readmission in the future.

## Methods

### Subjects selection

**Fig. 1:**
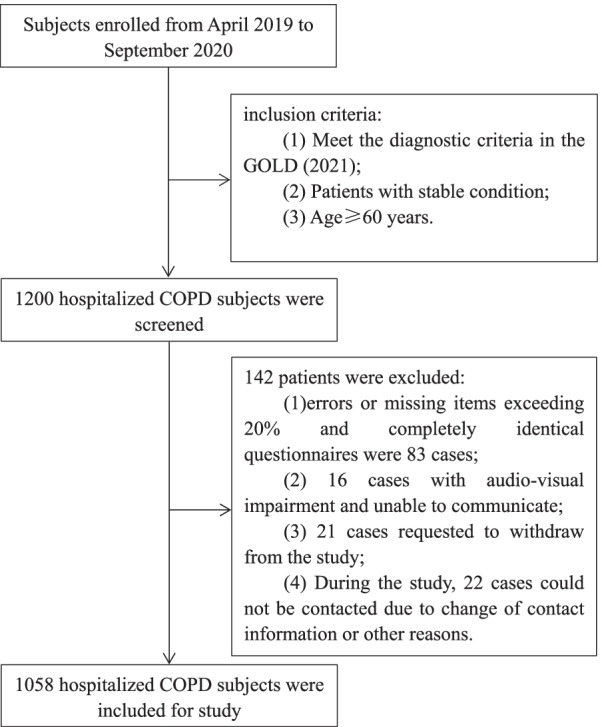
Flowchart of inclusion and exclusion criteria

From April 2019 to August 2020, COPD patients who met the inclusion and exclusion criteria in respiratory department of 13 general hospitals in Ningxia, China were investigated. Patients were followed up from May 2019 to September 2020. Ethics approval for the data collection and the use of clinical data in the study were obtained from the Ethics Committee of the General Hospital of Ningxia Medical University (2020-643), and all subjects signed informed consent. 1200 patients were enrolled. Inclusion criteria: (1) it meets the GOLD (2021) diagnostic criteria for COPD: that is, it has the following characteristics: ① Previous diagnosis of COPD; ② Smoking more than 20 cigarettes a day for more than 15 years; ③Have a long-term history of exposure to a large number of biofuels or occupational dust in an enclosed space; ④ Symptoms, chronic course and progressive aggravation above 40 years old; ⑤ Chronic cough or expectoration, and dyspnea after gradual activity; ⑥ Symptoms persist, with less daytime variation. After excluding the possibility of pulmonary tuberculosis, the peak expiratory flow rate (PEFR) was measured*.* The PEFR of 2 receptor agonist salbutamol 2 was improved by less than 20% after repeated determination 15 min after spray; FEV1/FVC0.7; (2) COPD atients with stable condition: the patient has stable or mild cough, expectoration, shortness of breath and other symptoms; (3) Age ≥ 60 years old. According to the following exclusion criteria, a total of 142 patients were excluded: (1) errors or missing items exceeding 20% and completely identical questionnaires were 83 cases; (2) 16 cases with audio-visual impairment and unable to communicate; (3) 21 cases requested to withdraw from the study; (4) During the study, 22 cases could not be contacted due to change of contact information or other reasons. A total of 1058 patients were included (Fig. 1).

### Research tools

General data questionnaire: designed by researchers according to the purpose and content of the research, through literature review and preliminary investigation, including age, sex, marital status, education level, smoking status, comorbidity, hospitalization times of acute exacerbation of COPD in the past 1 year, etc.

Modified Medical Research Council Dyspnea Scale (mMRC) are used to assess the severity of dyspnea in COPD patients, and verified to have good testing characteristics in Chinese population, it is divided into five levels. Level 0: difficulty breathing only during strenuous activity; Level 1: shortness of breath when walking briskly on flat ground or walking on a hill; Level 2: walking on flat ground slower than peers or needing to stop to rest due to shortness of breath; Level 3: walk on flat ground for about 100 m or need to stop for breath after a few minutes; Level 4: unable to leave the house because of severe breathing difficulties, or having difficulty breathing when putting on or undressing [13].

Activities of Daily Living (ADL) are used to evaluate patients’ ability of Daily Living Activities. It was first published in 1965 by Dorothy Barthel and Florence Mahone, and it was verified to have good test characteristics among Chinese people [14] includes 10 items. The total score is 100 points, and the evaluation standard is: 81~100 points, life completely self-care. 61~80 points, mild dysfunction, able to complete daily activities independently; 41~60 points, moderate dysfunction, need help in life ≤ 40 points, severely dysfunctional or totally dependent, most daily activities cannot be completed or require human care [15].

The Geriatric Depression Scale (GDS) is a measure of Depression in the elderly over the last 1 week. Prepared by Brink Equal in 1982, proved to have good test characteristics in The Chinese population [16] includes 30 entries. The total score is 30 and the assessment standard is: 0~10 is normal, that is no depression; 11 to 20 are classified as likely to have depressive symptoms; Between 21 and 30 is classified as depression [17].

The Mini Nutritional Assessment-Short Form (MNA-SF) is designed to evaluate Nutritional status. In 2001, it was proposed by Rubenstein et al. On the basis of Mini Nutritional Assessment and verified to have good testing characteristics in Chinese population [18] includes 8 questions. With a total score of 14, 12 to 14 is classified as normal nutritional status, 8 to 11 as at risk of malnutrition and 0 to 7 as undernourished [19].

COPD assessment test (CAT) scale used to assess the severity of COPD. Proposed by Jones on the basis of the St George Respiratory Questionnaire (SGRQ) in 2009, it has proven to have good test characteristics in a Chinese population [20] Includes 8 questions. The total score is 40 points, with the total score < 10 indicating mild illness, 10 < total score ≤ 20 indicating moderate illness, 20 < total score ≤ 30 indicating serious illness, and > 30 indicating very serious illness [21].

### Data collection methods

The researchers conducted a preliminary survey of 48 elderly patients with COPD and improved the questionnaire. The investigator of each hospital shall be trained uniformly (Investigators having worked in respiratory medicine department for 5 y or more, having bachelor’s degree and above with the qualification certificate of supervisor nurse, having questionnaire investigation experience), the training contents shall include the research purpose, research contents, questionnaire filling requirements, etc., trained investigator ask the patient about each item of the questionnaire and fill out each item according to the patient’s answers. Check if there is any missing item, take it back after checking. Patients were followed up to 30 days after discharge, including whether they were readmitted for acute exacerbation within 30 days after discharge, whether they took medication regularly and whether they took rehabilitation exercise, etc., and the readmission season of patients was recorded.

### Construction and verification of risk prediction model

A total of 28 variables were included in the screening of indicators for the construction of risk prediction model. After single factor analysis, a total of 15 variables such as age, education level and smoking status entered the regression analysis. Finally, 8 meaningful factors of logistic regression analysis were included in the construction of the model. The research objects are divided into training set (n = 741) and test set (n = 317) according to the ratio of 7:3. Based on the influencing factors, four kernel function models of SVM, linear SVM, polynomial SVM, sigmoid SVM and RBF SVM, are constructed. The prediction efficiency of the four kernel functions is analyzed and compared through the precision, recall, accuracy, F1 index and the area under the ROC curve (AUC) respectively.

### Data analysis

Epidata 3.1 was used for data entry, SPSS 25.0 and MATLAB R2020b were used for statistical analysis. Measurement data were described by median and quartile, while counting data were described by frequency and percentage. The measurement data of the two groups were compared by Mann–Whitney U test, the grading data were compared by Wilcoxon W test, and the counting data were compared by *χ*^2^ test. precision, recall, accuracy, F1 index and AUC evaluation model prediction ability. In all analyses, statistical significance was set at *P* < 0.05.

## Results

### General information of subjects

This study investigated 1058 elderly patients with COPD, including 645 males (60.96%) and 413 females (39.04%), aged from 60 to 96 years old. For other general information, see Table 1.

**Table 1:** Single factor analysis of different characteristics of the two groups

Characteristics	Non-readmission (*n* = 828)	Readmission (*n* = 230)	*χ*^2^/*Z* value	*P* value
Age (years)				
60~69	341 (41.18)	73 (31.74)	8.997*	0.011
70~79	349 (42.15)	103 (44.78)		
≥ 80	138 (16.67)	54 (23.48)		
Sex				
Male	509 (61.47)	136 (59.13)	0.415*	0.519
Female	319 (38.53)	94 (40.87)		
BMI				
< 18.5 kg/m^2^	141 (17.03)	37 (16.09)	5.330*	0.149
18.5~23.9 kg/m^2^	328 (39.61)	93 (40.43)		
24~27.9 kg/m^2^	245 (29.59)	56 (24.35)		
≥ 28 kg/m^2^	114 (13.77)	44 (19.13)		
Marital status				
Married	615 (74.28)	179 (77.83)	3.759*	0.153
Unmarried	62 (7.49)	21 (9.13)		
Widowed	151 (18.23)	30 (13.04)		
Way of living				
Not live alone	746 (90.10)	210 (91.30)	0.301*	0.583
Living alone	82 (9.90)	20 (8.70)		
Education level				
Illiteracy	340 (41.06)	118 (51.30)	16.568*	0.002
Primary	243 (29.35)	73 (31.74)		
Junior high school	153 (18.48)	23 (10.00)		
High school or technical secondary school	72 (8.69)	14 (6.09)		
Junior college or above	20 (2.42)	2 (0.87)		
Professional				
History of contamination	26 (3.14)	5 (2.17)	0.591*	0.442
No contamination	802 (96.86)	225 (97.83)		
Smoking status				
Never smoker	489 (59.06)	115 (50.00)	46.789*	< 0.001
Former smoker	300 (36.23)	73 (31.74)		
Current smoker	39 (4.71)	42 (18.26)		
Number of comorbid				
≤ 2	684 (82.61)	189 (82.17)	0.024*	0.878
> 2	144 (17.39)	41 (17.83)		
Hypertension				
Yes	359 (43.36)	113 (49.13)	2.428*	0.119
No	469 (56.64)	117 (50.87)		
Diabetes				
Yes	160 (19.32)	65 (28.26)	8.587*	0.003
No	668 (80.68)	165 (71.74)		
CHD				
Yes	207 (25.00)	39 (16.96)	6.526*	0.011
No	621 (75.00)	191 (83.04)		
Hypoproteinemia				
Yes	14 (1.69)	7 (3.04)	1.693*	0.193
No	814 (98.31)	223 (96.96)		
Hospitalization times of acute exacerbation of COPD in the past 1 year				
< 2	500 (60.39)	87 (37.83)	37.092*	< 0.001
≥ 2	328 (39.61)	143 (62.17)		
Seasonal factors				
Spring	163 (19.68)	55 (23.91)	42.738*	< 0.001
Summer	205 (24.76)	21 (9.13)		
Fall	232 (28.02)	50 (21.74)		
Winter	228 (27.54)	104 (45.22)		
Long-term home oxygen therapy				
Yes	250 (30.19)	46 (20.0)	9.282*	0.002
No	578 (69.81)	184 (80.0)		
Regular medication				
Yes	213 (25.72)	34 (14.78)	12.043*	0.001
No	615 (74.28)	196 (85.22)		
Rehabilitation exercise				
Yes	276 (33.33)	51 (22.17)	10.497*	0.001
No	552 (66.67)	179 (77.83)		
PaCO_2_ (mmHg)	41.15 (36.00, 48.00)	41.90 (36.00, 49.05)	− 0.878^#^	0.380
PaO_2_ (mmHg)	66.35 (58.78, 77.00)	65.90 (59.90, 81.25)	− 0.277^#^	0.782
SaO_2_ (%)	92 (90, 94)	92 (90, 94)	− 0.489^#^	0.625
Respiration rate (times/minute)	21 (20, 22)	21 (20, 22)	− 0.774^#^	0.439
Course of disease (years)	4 (1, 10)	6 (3, 10.50)	− 3.827^#^	< 0.001
ADL				
Completely independent	142 (17.15)	16 (6.96)	− 2.963*	0.003
Mild dysfunction	482 (58.21)	133 (57.83)		
Moderate dysfunction	137 (16.55)	47 (20.43)		
Severe dysfunction or complete dependence	67 (8.09)	34 (14.78)		
GDS				
There is no depression	376 (45.41)	77 (33.48)	− 2.904*	0.004
Mild depression	356 (43.00)	96 (41.74)		
Moderate to severe depression	96 (11.59)	57 (24.78)		
mMRC				
Level 0	97 (11.71)	8 (3.48)	− 4.141*	< 0.001
Level 1	208 (25.13)	36 (15.65)		
Level 2	297 (35.87)	51 (22.17)		
Level 3	185 (22.34)	85 (36.96)		
Level 4	41 (4.95)	50 (21.74)		
MNA-SF				
Normal nutritional status	268 (32.37)	20 (8.70)	− 8.568*	< 0.001
Risk of malnutrition	386 (46.62)	87 (37.83)		
Malnutrition	174 (21.01)	123 (53.49)		
CAT				
Slight symptoms	26 (3.14)	11 (4.78)	− 1.713*	0.087
Medium	287 (34.66)	72 (31.30)		
Serious	426 (51.45)	127 (55.22)		
Very serious	89 (10.75)	20 (8.70)		

*BMI* Body mass index, *CHD* coronary heart disease, *ADL* activities of daily living, *GDS* the Geriatric Depression Scale, *mMRC* Modified Medical Research Council Dyspnea Scale, *MNA-SF* the Mini Nutritional Assessment-Short Form, *CAT* COPD assessment test

*Is the *χ*^2^ test; ^#^is the Mann–Whitney U test; *is the Wilcoxon W test

### Risk prediction model construction index screening

Single factor analysis showed that there were statistically significant differences in age, education level, smoking status, diabetes, coronary heart disease (CHD), hospitalization times of acute exacerbation of COPD in the past 1 year, seasonal factors, whether Long-term home oxygen therapy, whether regular medication, whether rehabilitation exercise, course of disease, ADL, GDS, mMRC and MNA-SF of the subjects (*P* < 0.05), (Table1).

In Single factor analysis, statistically significant factors were independent variables, whether readmission was the dependent variable, was included in binary Logistic regression analysis. The results showed that education level, smoking status, CHD, hospitalization times of acute exacerbation of COPD in the past year, whether Long-term home oxygen therapy, whether regular medication, MNA-SF and seasonal factors were the influencing factors (*P* < 0.05), (Table 2). The eight significant factors of Logistic regression analysis were incorporated into the model construction.

**Table 2:** Logistic regression of acute exacerbation readmission in elderly patients with COPD within 30 days

Influencing factors	*Β* value	*SE* value	Wald value	*P* value	OR value	95%CI
Age	0.156	0.179	0.761	0.383	1.169	0.823~1.660
Education level	− 0.358	0.140	6.499	0.011	0.699	0.531~0.921
Smoking status	0.960	0.198	23.516	0.000	2.612	1.772~3.851
Diabetes	0.642	0.413	2.416	0.120	1.900	0.846~4.268
CHD	1.030	0.357	8.334	0.004	2.801	1.392~5.637
Hospitalization times of acute exacerbation of COPD in the past 1 year	1.359	0.267	25.872	0.000	3.891	2.305~6.567
Seasonal factors	–	–	16.110	0.001	–	–
Spring	1.339	0.427	9.833	0.002	3.814	1.652~8.806
Fall	1.184	0.424	7.802	0.005	3.268	1.424~7.502
Winter	1.623	0.409	15.740	0.000	5.070	2.274~11.306
Long-term home oxygen therapy	− 1.125	0.334	11.305	0.001	0.325	0.169~0.626
Regular medication	− 1.161	0.357	10.596	0.001	0.313	0.156~0.630
Rehabilitation exercise	− 0.494	0.298	2.749	0.097	0.610	0.340~1.094
Course of disease	0.006	0.015	0.150	0.699	1.006	0.977~1.035
ADL	− 0.228	0.192	1.409	0.235	0.796	0.547~1.160
GDS	0.119	0.189	0.393	0.531	1.126	0.777~1.631
mMRC	0.021	0.134	0.026	0.873	1.022	0.786~1.327
MNA-SF	1.541	0.224	47.241	0.000	4.671	3.010~7.249
Constant	− 9.085	1.084	70.301	0.000	0.000	**–**

*CHD* coronary heart disease, *ADL* activities of daily living, *GDS* the Geriatric Depression Scale, *mMRC* Modified Medical Research Council Dyspnea Scale, *MNA-SF* the Mini Nutritional Assessment-Short Form

### Construction and verification of risk prediction model

Eight significant factors were included in logistic regression analysis to build a SVM model, the subjects were divided into training set (n = 741) and test set (n = 317) in a 7:3 ratio.

Since the dimensions of variables are inconsistent and the range of values is large, in order to avoid tedious calculation and ignore small numerical data, SVM is applied to normalize the data to [− 1, 1] before modeling. Using grid search and cross-validation methods to find the optimal *(c*, *g*) pairs, The optimal (*c*, *g*) was used to establish the SVM model using Linear, Polynomial, Sigmoid and RBF kernel functions respectively.

### Comparison of predictive performance of risk prediction model

**Fig. 2:**
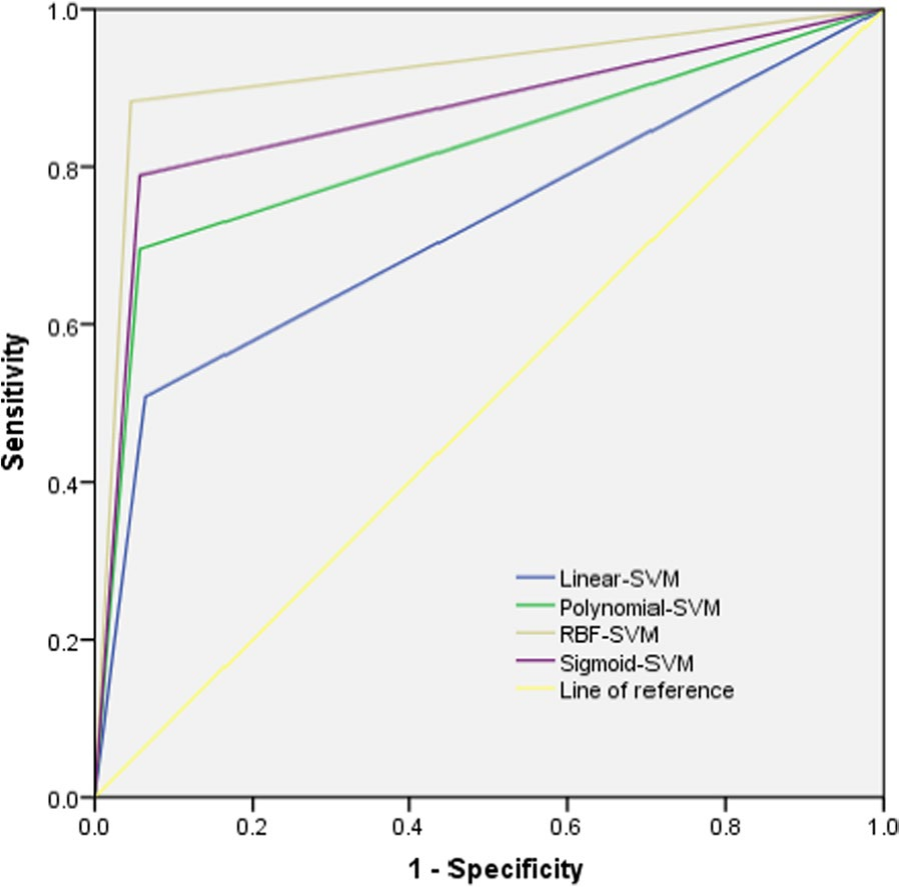
ROC of the training sets of four functions

**Fig. 3:**
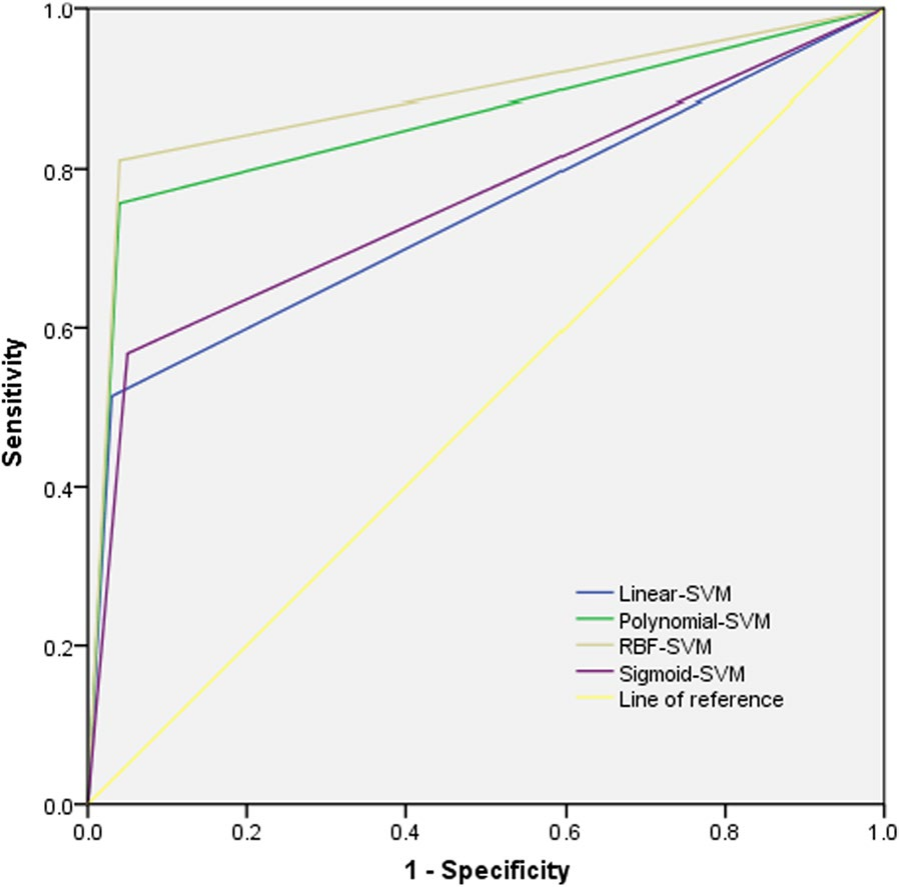
ROC of the test sets of four functions

The prediction effect was evaluated by precision, recall, accuracy, F1 index and AUC, In the training set and test set, the precision, recall, accuracy, F1 index and AUC of RBF-SVM model are better than those constructed by Linear-SVM, Polynomial-SVM and Sigmoid-SVM, shown in Table 3, Figs. 2 and 3.

**Table 3:** Predictive performance indicators of the four kernel functions

Evaluation index	Training set				Test set			
	Linear-SVM	Polynomial-SVM	Sigmoid-SVM	RBF-SVM	Linear-SVM	Polynomial-SVM	Sigmoid-SVM	RBF-SVM
Precision (%)	69.89	78.07	79.37	84.21	86.36	87.50	80.77	88.24
Recall (%)	50.78	69.53	78.74	88.19	51.35	75.68	56.76	81.08
Accuracy (%)	83.92	88.69	90.81	93.82	85.11	90.78	85.11	92.20
F1 index	0.59	0.74	0.79	0.86	0.64	0.81	0.67	0.85
AUC	0.722	0.819	0.866	0.918	0.742	0.858	0.759	0.885

*AUC* area under the ROC curve, *SVM* support vector machine

## Discussion

Studies have shown that acute exacerbations of COPD occur repeatedly in the course of disease development and may lead to readmission due to acute exacerbations within a short period after discharge. Acute exacerbation readmission within 30 days due to the short acute exacerbation cycle, not only severely damages lung function and increases the risk of death, but also occupies a large number of medical resources [4]. Based on the SVM model, this study used Linear-SVM, Polynomial-SVM, Sigmoid-SVM and RBF-SVM kernel function to develop and verify the risk prediction model of acute exacerbation readmission within 30 days of elderly patients with COPD. The 28 variables were analyzed, and 8 factors that were significant for logistic regression analysis were finally included in the model construction, namely education level, smoking status, CHD, the number of hospitalizations for acute exacerbation of COPD in the past 1 year, seasonal factors, home oxygen therapy, regular medication, nutritional status. The prediction model has good prediction performance on both the training set and the test set, and the RBF kernel function fitting model in both the training set and the test set has the best effect. This result is consistent with the comparison results of the four kernel functions in the study of Du et al.^12^. This may be related to the characteristics of the RBF kernel function itself. The RBF kernel function determines the classification boundary according to the distance of each support vector, and is the most flexible method. The eight variables required to construct the predictive model are readily available in the follow-up of elderly patients with COPD, suggesting that the model may be a convenient tool for predicting the risk of readmission for acute exacerbation within 30 days in elderly patients with COPD, it can help medical staff to effectively assess and manage the risk of acute exacerbation, slow the disease process and reduce mortality.

In this study, educational level and smoking status were the influencing factors of readmission for acute exacerbation. Collinsworth et al. [22] showed that a high level of self-management education was a protective factor for readmission. In this study, patients with higher education level (*β* = − 0.358) had lower risk of acute exacerbation and readmission, which may be related to the fact that patients with higher education level have a stronger ability to understand and comprehend health education knowledge, and can pass various media and get more knowledge about COPD prevention and treatment through the Internet. Smoking is significantly associated with readmission for acute exacerbations. Four studies included 21,270 subjects, and the 52-week observation found that smokers had a higher risk of readmission than quit smoking and never-smokers. In addition, this study also found that current or heavy smokers had a higher frequency of exacerbations than former or light smokers [RR: (0.81–0.99): (0.92–1.29)], which may smoking counteracts the therapeutic effect of inhaled glucocorticoids and can aggravate the patient's inflammatory state, causing greater damage to lung function [22]. Therefore, for patients with low education level, health education should be carried out in an easily acceptable way such as video broadcasting, and inform the smoking patients of the impact of smoking on the disease and the harm to the body.

CHD, the number of hospitalizations for acute exacerbation of COPD in the past 1 year, and seasonal factors were the influencing factors of readmission for acute exacerbation. COPD often coexists with other diseases. The most common one is cardiovascular disease represented by CHD. Patients with CHD have more severe shortness of breath and lower exercise tolerance after exercise [23]. In addition, this study found that patients with ≥ 2 hospitalizations for acute exacerbations in the past 1 year were 3.891 times more likely to have readmissions than patients with < 2. Studies by other scholars have also confirmed that the number of exacerbations in COPD patients in the past year can predict the risk of early readmission, and regardless of the severity of the disease, the history of previous exacerbations is the best predictor of later exacerbations [24]. The acute exacerbation of COPD patients is usually caused by viral/bacterial infection, which not only increases the risk of readmission for acute exacerbation, but also leads to the decline of lung function and quality of life, and the increase of mortality. Therefore, the prevention of acute exacerbations of COPD is particularly important. Medical staff should conduct health education for patients with acute exacerbation hospitalization ≥ 2 times in the past year to help them master the knowledge of pulmonary rehabilitation. Study has shown that acute exacerbation of COPD is closely related to seasonal changes. Patients with low temperature have obvious immune dysfunction, and chronic airway inflammation is likely to aggravate after cold air stimulation [25]. In addition, COPD patients are mostly elderly, their airway reactivity is increased, they are more sensitive to changes in air temperature, and are likely to cause airway spasm [23]. This study found that the readmission rates of elderly COPD patients in spring, summer, autumn, and winter were 20.60%, 21.36%, 26.66%, and 31.38%, respectively. The risk of readmission in spring and winter was 3.814 times and 5.070 times that in summer, respectively, similar to the results of a study by Fang Jiaying [26].

Home oxygen therapy, regular medication and nutritional support are the influencing factors of readmission for acute exacerbation. Long-term home oxygen therapy, as one of the treatment measures in the stable management of COPD patients, can delay the progression of the disease. In addition, COPD is mainly treated with drugs for a long time. At present, there are many kinds of drugs for the treatment of COPD, and elderly patients have poor memory and poor medication compliance. In this study, only 23.35% of the patients took regular medication as prescribed by the doctor. The multivariate analysis showed that the risk of readmission was 0.313 times higher than that of regular users, which was consistent with the results of Kume [27]. This may be related to the fact that regular medication can improve the pulmonary ventilation function of patients, effectively prevent the development of the disease, and thus reduce the number of hospitalizations for acute exacerbations. COPD is a chronic wasting disease. It is a common disease that causes malnutrition due to increased energy consumption, electrolyte disturbances, digestive disturbances and the influence of drugs [28–30]. In this study, the risk of malnutrition and the incidence of malnutrition in elderly patients with COPD were 44.71% and 28.07%, respectively. The results of multivariate analysis showed that patients with poorer nutritional status (*β* = 1.541) had a higher risk of readmission, which was similar to the findings of Wu et al. [31]. This may be related to muscle atrophy caused by poor nutritional status, resulting in weakened respiratory muscle strength, further affecting respiratory function, and promoting acute exacerbation of COPD.

Although this study based on the SVM model predicts the 30-day acute exacerbation readmission risk in elderly COPD patients with good performance on both the training set and the test set, there are still some limitations. First of all, the data of the training set and test set of this study are from the same sample group, which may limit its promotion in different populations, hospitals and regions. Second, because C-reactive protein and sputum culture results related to COPD are not routine inspection items in some surveyed hospitals, relevant indicators were not included in this study, which may lead to bias in the results, and relevant indicators can be added for analysis in the future.

## Conclusions

In summary, this study found factors that may affect readmission. The SVM model constructed based on the above factors has achieved a certain predictive effect on predicting the risk of readmission of patients, and has a certain reference value, which can provide reference information for risk assessment and clinical prevention and treatment of readmission.

## Data Availability

The datasets used and/or analyzed during the current study are available from the author of ZhangRui on reasonable request.

## Abbreviations

COPD: Chronic obstructive pulmonary disease
SVM: Support vector machine
AUC: Area under the ROC curve
mMRC: Modified Medical Research Council Dyspnea Scale
ADL: Activities of daily living
GDS: The Geriatric Depression Scale
MNA-SF: The Mini Nutritional Assessment-Short Form
CAT: COPD assessment test
CHD: Coronary heart disease
BMI: Body mass index
GOLD: The Global Initiative on Chronic Obstructive Pulmonary Disease
